# Influence of Genetic Variations on Levels of Inflammatory Markers of Healthy Subjects at Baseline and One Week after Clopidogrel Therapy; Results of a Preliminary Study

**DOI:** 10.3390/ijms140816402

**Published:** 2013-08-08

**Authors:** Payman Shahabi, Gérard Siest, Bernard Herbeth, Daniel Lambert, Christine Masson, Jean-Sébastien Hulot, Sébastien Bertil, Pascale Gaussem, Sophie Visvikis-Siest

**Affiliations:** 1 UMR INSERM U 1122, IGE-PCV, Faculté de Pharmacie, Université de Lorraine, 30 Rue Lionnois, Nancy 54000, France; E-Mails: payman.shahabi@inserm.fr (P.S.); gerard.siest@univ-lorraine.fr (G.S.); bernard.herbeth@inserm.fr (B.H.); daniel.lambert@inserm.fr (D.L.); christine.masson@univ-lorraine.fr (C.M.); 2 INSERM UMR S956, Université Pierre et Marie Curie, Paris 75005, France; E-Mail: jean.hulot@mssm.edu; 3 AP-HP, Hôpital Européen Georges Pompidou, Paris 75908, France; E-Mails: sbastienbertil@yahoo.fr (S.B.); pascale.gaussem@egp.aphp.fr (P.G.); 4 Sorbonne Paris Cité, Université Paris Descartes, Paris 75270, France; 5 INSERM UMR S765, Faculté de Pharmacie, Université Paris V, Paris 75006, France

**Keywords:** inflammation, cytochrome P450, polymorphism, epoxyeicosatrienoic acids, clopidogrel, C-reactive protein, haptoglobin

## Abstract

We aimed to assess the association between the most common polymorphisms of cytochrome P450 (CYP) epoxygenases on the plasma levels of inflammatory markers in a population of healthy subjects. We also sought to determine whether CYP2C19*2 polymorphism is associated with the anti-inflammatory response to clopidogrel. In a population of 49 healthy young males, the baseline plasma levels of inflammatory markers including C-reactive protein, haptoglobin, orosomucoid acid, CD-40 were compared in carriers *vs*. non-carriers of the most frequent CYP epoxygenase polymorphisms: CYP2C9*2, CYP2C9*3, CYP2C19*2, CYP2C8*2 and CYP2J2*7. Also, the variation of inflammatory markers from baseline to 7 days after administration of 75 mg per day of clopidogrel were compared in carriers *vs*. non-carriers of CYP2C19* allele and also in responders *vs*. hypo-responders to clopidogrel, determined by platelet reactivity tests. There was no significant association between epoxygenase polymorphisms and the baseline levels of inflammatory markers. Likewise, CYP2C19* allele was not associated with anti-inflammatory response to clopidogrel. Our findings did not support the notion that the genetic variations of CYP epoxygenases are associated with the level of inflammatory markers. Moreover, our results did not support the hypothesis that CYP2C19*2 polymorphism is associated with the variability in response to the anti-inflammatory properties of clopidogrel.

## 1. Introduction

Our knowledge of the contribution of inflammation to the pathophysiology of cardiovascular disorders has progressed to the point that, currently, coronary artery disease (CAD) and arterial hypertension (HTN) are considered to be diseases of inflammation characterized by interaction among platelets, leukocytes and endothelial cells [1–3].

Cytochrome P450 (CYP) enzymes play an indispensable role in the regulation of inflammation through the biosynthesis of endobiotics that modulate inflammation, such as arachidonic acid (AA) derivatives epoxyeicosatrienoic acids (EETs). Indeed, the main human CYP epoxygenases, *i.e*., CYP2C8, CYP2C9, CYP2C19 and CYP2J2, catalyse the epoxidation of AA to EETs that have been shown to display potent anti-inflammatory properties [4,5]. The genes encoding the epoxygenases are polymorphic; therefore, it is logical to hypothesize that the CYP epoxygenase loss-of-function polymorphisms are associated with a decrease in the relative abundance and also the inflammation-reducing properties of EETs. The first part of the present study was designed to analyze plasma levels of a selected number of inflammatory markers as a function of the most frequent epoxygenase gene polymorphisms, in a population of young healthy male volunteers.

CYPs also play central roles in the metabolism and disposition of a wide range of drugs and xenobiotics with potential anti-inflammatory effects such as clopidogrel [6]. Clopidogrel is the thienopyridine of choice for dual antiplatelet therapy in patients with acute coronary syndrome (ACS) and those treated with percutaneous coronary intervention (PCI) [7]. Clopidogrel is a pro-drug that is required to be converted to its active metabolite through a two-step oxidative process. Both steps involve several hepatic CYP isoenzymes, including CYP2C19, CYP3A4/5, CYP2C9, CYPP1A2 and CYP2B6 [8]. The ability of clopidogrel to inhibit platelet-mediated thrombogenicity is partly influenced by the polymorphisms of the clopidogrel-CYP-metabolizing enzymes and also the P2Y12 receptor gene polymorphism [9,10]. We originally reported that the CYP2C19*2 loss-of-function allele is involved in a reduced response to clopidogrel in healthy subjects [11]. In our small population of healthy volunteers, neither the polymorphism of CYP3A4 gene (IVS10 + 12G > A), nor the P2Y12 receptor gene polymorphism (*H2* allele) were associated with the platelet response to clopidogrel, although there was a slight trend for *H2H2* homozygous subjects to being less responders to clopidogrel [12,13]. Being primarily a platelet aggregation inhibitor, clopidogrel has also been shown to exhibit pleiotropic anti-inflammatory properties [6]. However, the inflammation-reducing effects of clopidogrel have not been a persistent finding in all trials and the results of different studies in this field are sometimes contradictory [14–19]. We hypothesized that the gene variations associated with a poor responsiveness to antiplatelet effects of clopidogrel could also contribute to the variability in response to its potential anti-inflammatory properties. Accordingly, the second aim of this study was to investigate whether the anti-inflammatory effect of standard-dose clopidogrel is affected by the functional variants of CYP2C19 and P2Y12 genes (CYP2C19*2 and *H2* alleles) in healthy subjects taking clopidogrel.

## 2. Results and Discussion

Demographic and baseline biochemical characteristics of subjects are presented in Table 1. In order to minimize the influence of female hormones and to be free from the inflammatory response that is known to occur during CAD and the interventional procedures and also to eliminate the likely anti-inflammatory effects of other medications, this study included only healthy male volunteers.

Table 2 presents the plasma concentrations of the inflammatory markers in carriers and non-carriers of the AA-metabolizing CYP polymorphisms. There was no significant association between plasma biomarker levels and CYP2C19*2, CYP2J2*7, CYP2C8*3, CYP2C9*2 and CYP2C9*3 polymorphisms in the healthy population analyzed.

Our findings did not provide support to the hypothesis that, under physiological conditions, the CYP epoxygenase loss-of-function polymorphisms are associated with an inter-individual variability in the plasma levels of systemic inflammatory markers. However, it should be considered that the examination of this hypothesis in a population of young healthy individuals with the already low levels of circulating inflammatory markers might not be straightforward. Also, we acknowledge that our study was performed on a small population size and a lack of power cannot be excluded. Accordingly, further larger well-designed studies are necessary to better assess the potential associations between the most common epoxygenase polymorphisms and the inflammatory markers in healthy individuals. Furthermore, due to small sample size, we failed to investigate if the co-existence of multiple epoxygenase defective alleles is associated with the levels of markers of inflammation in healthy subjects; this theory also requires to be tested by the future studies.

Our observations in this study are, somehow, consistent with those of our previous study in which, we investigated whether CYP2C19*2 polymorphism is associated with inflammatory markers of a healthy population, consisting of 178 men and 181 women. In that study we found that the plasma level of hs-CRP is significantly higher in healthy females carriers of CYP2C19*2 allele, but not in males [20]. In the present study, we observed that not only CYP2C19*2, but also other CYP epoxygenase polymorphisms are not associated with levels of systemic inflammatory markers of healthy males.

Additionally, our findings are also consistent with those of Hoffmann *et al*. [21], who failed to show statistically significant relationship between CYP2J2*7 polymorphism and the levels of hs-CRP, fibrinogen and leukocyte count in a large number of patients with CAD. Likewise, the results of a large population-based prospective study by Kaur-Knudsen *et al*. [22], showed that there is not a significant association between CYP2C9*2 and CYP2C9*3 polymorphisms and the level of CRP.

In regard to the variation of inflammatory marker levels in response to clopidogrel, in general and regardless of the genetic polymorphisms, clopidogrel administration was not associated with changes in the inflammatory marker levels (Table 3). In addition, no significant association was found between the changes of inflammatory markers and either CYP2C19*2, or *P2Y12 H2* Polymorphisms (Table 3). Also, the other studied CYP polymorphisms, including CYP2C8*3, CYP2C9*2 and CYP2C9*3, were not associated with the difference of the levels of markers from baseline to 7 days after taking clopidogrel (data not presented). Likewise, as presented in Table 4, no significant difference in the variation of plasma biomarkers was observed between responders *vs*. hypo-responders to clopidogrel, at baseline and one week after clopidogrel intake.

Clopidogrel is currently the most widely prescribed platelet P2Y12 receptor inhibitor [23]. However, there is a significant inter-individual variability in the response to the antiplatelet effect of clopidogrel, with up to 40% of patients being classified as poor responders to this drug [24–27]. This variability is partly attributed to the loss-of-function CYP2C19*2 and, in some studies and to lesser extent, to the *P2Y12 H2* polymorphisms. Since, in addition to thrombus formation, platelet has a central role to the initiation and maintenance of inflammation [28], it not surprising to observe that clopidogrel has also pleiotropic anti-inflammatory properties [6]. The inflammation-reducing effects of clopidogrel could be clinically beneficial since on-clopidogrel patients with higher levels of inflammatory markers have been demonstrated to have an increased incidence of adverse cardiovascular events [29]. However, controversy has surrounded the anti-inflammatory of clopidogrel [14–19]. Since the genetic variations of applicable genes (especially CYP2C19 and P2Y12) have been shown to be the main culprit in the variability in the responsiveness to the clopidogrel, it is logical to speculate that the same relevant polymorphisms could lead to an inter-individual variability in response to anti-inflammatory effects of clopidogrel.

In general, our results did not provide evidence that clopidogrel exhibits anti-inflammatory effects in healthy male individuals. Yet, it should be considered that since the recruited individuals in our study have been all from young and healthy people, the concentrations of inflammatory markers in their plasma, even at baseline and before taking clopidogrel, were relatively low; the interpretation of results in this situation might be inconclusive. Our findings in the current study are inconsistent with those of Klinkhardt *et al*. [30], who showed that clopidogrel could attenuate significantly inflammation in 10 healthy subjects. Possible sources of discrepancy would be small sample sizes of the both studies and the differences in the determined inflammatory markers and study settings. Therefore, further well-conducted investigations with enough sample size are warranted to assess the pleiotropic anti-inflammatory effects of clopidogrel. Additionally, our current findings did not support the theory that either the CYP2C19*2 or the P2Y12 H2 alleles influence the anti-inflammatory potential of clopidogrel in healthy subjects. Nevertheless, here again, the low baseline levels of plasma inflammatory markers in our young healthy population may make interpretation of the results indecisive and unreliable. Also, since in this study the subjects were not genotyped for the gain-of-function CYP2C19 polymorphism (CYP2C19*17), it should be acknowledged that those who have been categorized under CYP2C19*1/*1 genotype group (also known as extensive metabolizers of clopidogrel), might also include the carriers of CYP2C19*17 allele who are ultra-metabolizers of clopidogrel.

Also, certain clopidogrel-metabolizing CYPs, specifically CYP2C9 and CYP2C19 are also involved in the metabolic transformation of AA to EETs. Therefore, another hypothesis that may explain our findings is that clopidogrel might act as a competitive inhibitor of CYP2C9/CYP2C19 resulting in a decrease in the production and in the anti-inflammatory effects of EETs in on-clopidogrel patients. In addition, clopidogrel has been shown to be a moderate inhibitor of CYP2C8 [31], a main human EET-producing epoxygenase, resulting in a decrease in the EET formation and in the EET-induced inflammation reduction. Together, as a paradigm of a xenobiotic-endobiotic interaction, the likely clopidogrel-EET interaction needs further studies to elucidate its clinical implication.

Last but not least, it should be pointed out that the current study, which has been performed exclusively on healthy males, is the pilot study of larger research projects carried out on post-PCI and hypertensive patients, including both males and females. The results of these studies will be released in the near future.

## 3. Experimental Section

### 3.1. Subjects and Study Protocol

The study design and the inclusion criteria for the subjects were described in details elsewhere [13,32]. Briefly, forty nine healthy male Caucasians (aged 18–35 years) who met the following conditions were enrolled: non-smokers, normal physical and laboratory results, no history of use of medications as well as normal platelet count, normal maximal platelet aggregation in response to platelet agonists and normal PFA-100^®^ assay. The subjects were given a standard dose of clopidogrel 75 mg once daily for 7 consecutive days in the presence of medical staff. For each subject, the plasma concentrations of a selected number of inflammatory proteins including high-sensitivity C-reactive protein (hs-CRP), haptoglobin (Hp), orosomucoid acid (Oroso) and CD-40 were compared before and 7 days after clopidogrel administration. In addition, the responsiveness to the antiplatelet effect of clopidogrel (responders *vs*. hypo-responders), was compared, in each participant, at day 1 (baseline) and 7 of clopidogrel intake using the ADP-induced platelet aggregation as well as the phosphorylated vasodilator-stimulated phosphoprotein (VASP).

Additionally, at baseline, and before taking clopidogrel, the levels of inflammatory markers were compared across the population, in the function of the most frequent CYP epoxygenase gene polymorphisms in Caucasians: CYP2C8*3 (rs10509681), CYP2C9*2 (rs1799853), CYP2C9*3 (rs1057910), CYP2C19*2 (rs4244285) and CYP2J2*7 (rs890293). Also, the changes in the levels of inflammatory markers, from baseline to end of clopidogrel therapy, were compared between the carriers *vs*. non-carriers of CYP2C19*2 and *P2Y12 H2* alleles and also between responders *vs*. non-responders to clopidogrel.

The study was approved by the Committee for the Protection of Human Subjects in Biomedical Research, which functions for the Cochin University Hospital (Paris, France), and all the subjects gave their written informed consent to participate.

### 3.2. Genotyping and Protein Quantification

Genotyping was performed by LGC Genomics and KBioscience Laboratory using KASP™ genotyping assay. The primers used for genotyping are presented in Table 5.

The plasma levels of CRP, Hp and Oroso were immunonephelometrically determined, using BN II (Siemens Healthcare Diagnostics, Marburg, Germany); the intra-assay variation coefficient for CRP, Hp and Oroso was 2.6%, 3.2% and 2.6%, respectively. Moreover, soluble CD-40 was measured using commercial enzyme-linked immunosorbent assay kit according to the manufacturer’s instruction.

### 3.3. Platelet Function Studies

Platelet aggregation studies and the determination of the level of phosphorylated VASP were performed as detailed elsewhere [13,33]. Clopidogrel hypo-responsiveness was defined as a maximal 10 μM ADP-induced aggregation at 7 day above 50% and/or a VASP index above 60% after one week 75 mg/d clopidogrel administration [33].

### 3.4. Statistical and Data Analysis

ANOVA with repeated measures was used to test the 7-day changes of inflammatory markers, the average effect of polymorphisms and the polymorphisms/7-day changes interaction. Data were log transformed for CRP concentrations. Results are presented as arithmetic means (SD) or geometric means (range of 1 SD). Statistical analyses were performed using SAS 9.3 software (SAS Institute, Cary, Cary, NC, USA). Statistical significance was set at *p* ≤ 0.05.

## 4. Conclusions

Our findings did not support the hypothesis that, under physiological conditions, the most common polymorphisms of the main human CYP epoxygenase polymorphisms are associated with altered plasma level of inflammatory proteins. Moreover, we did not find a significant association between either the CYP2C19*2/*P2Y12 H2* polymorphisms or the antiplatelet responsiveness to clopidogrel and the inflammation-reducing properties of clopidogrel in healthy male subjects.

## Figures and Tables

**Table 1:** Patients’ characteristics.

Variables	General population (*n* = 49)
Demographic characteristics	
Age (year) †	25.8 ± 3.8
Male sex, (%)	49 (100)
Body mass index (kg/m^2^) †	23.2 ± 2.11

Vital signs	
Pulse rate (n) †	66.9 ± 9.4
Systolic blood pressure (mmHg) †	121.7 ± 8.4
Diastolic blood pressure (mmHg) †	68.6 ± 6.8

Hemogram	
White blood cells (×10^3^/μL) †	5.41 ± 1.13
Red blood cells (×10^6^/μL) †	4.72 ± 0.25
Hemoglobin (g/L) †	14.21 ± 0.55
Hematocrit (%) †	41.66 ± 1.60
Platelet (×10^3^/μL) †	220.72 ± 37.32

Biochemistry	
Glucose (mmol/L) †	4.76 ± 0.38
Aspartate aminotransferase (U/L) †	20.00 ± 5.97
Alanine aminotransferase (U/L) †	25.93 ± 10.19
Alkaline phosphatase (U/L) †	55.37 ± 11.39
Gamma-glutamyltransferase (U/L) †	20.38 ± 8.23
Creatinine (μmol/L) †	83.56 ± 11.05
Fibrinogen (g/L) †	2.28 ± 0.35

† Mean ± SD;

SD: standard deviation.

**Table 2:** Baseline inflammatory marker levels 1 in function of cytochrome P450 (CYP) epoxygenase polymorphisms.

Allele	Orosomucoid (g/L)	Haptoglobin (g/L)	CRP (mg/L)	CD40 (ng/L)
CYP2C19*2 (681 G > A); rs4244285				

CYP2C19*1/*1 (*n* = 30)	0.58 (0.10)	0.74 (0.28)	0.32 (0.16–0.65)	382 (153)
CYP2C19*1/*2 (*n* = 19)	0.62 (0.13)	0.85 (0.31)	0.31 (0.14–0.67)	323 (66)

CYP2J2*7 (−50 G > A); rs890293				

CYP2J2*1/*1 (*n* = 42)	0.60 (0.12)	0.79 (0.31)	0.38 (0.32–0.44)	359 (132)
CYP2J2*1/*7 (*n* = 7)	0.57 (0.09)	0.78 (0.34)	0.32 (0.15–0.69)	351 (96)

CYP2C8*3 (1196 A > G); rs10509681				

CYP2C8*1/*1 (*n* = 36)	0.60 (0.12)	0.82 (0.31)	0.33 (0.15–0.72)	328 (63)
CYP2C8*1/*3 (*n* = 13)	0.58 (0.10)	0.76 (0.27)	0.27 (0.17–0.43)	426 (190)

CYP2C9*2 (430 C > T); rs1799853				

CYP2C9*1/*1 (*n* = 37)	0.60 (0.12)	0.80 (0.32)	0.34 (0.16–0.76)	341 (97)
CYP2C9*1/*2 (*n* = 12)	0.59 (0.10)	0.84 (0.26)	0.26 (0.16–0.41)	424 (191)

CYP2C9*3 (1075 A > C); rs1057910				

CYP2C9*1/*1 (*n* = 42)	0.60 (0.12)	0.80 (0.30)	0.30 (0.15–0.57)	361 (133)
CYP2C9*1/*3 (*n* = 7)	0.62 (0.06)	0.88 (0.37)	0.73 (0.21–2.48)	366 (6)

1 Arithmetic mean (SD) or Geometric mean (range of 1 SD).

**Table 3:** Variation of inflammatory marker levels 1 in response to clopidogrel in the whole population and in function of CYP2C19*2 and *P2Y12 H2* Polymorphisms.

	Orosomucoid (g/L)		Haptoglobin (g/L)		CRP (mg/L)		CD40 (ng/L)	
				
	Day 1	Day 7	Day 1	Day 7	Day 1	Day 7	Day 1	Day 7
Whole population (*n* = 49)	0.61 (0.12)	0.60 (0.19)	0.78 (0.29)	0.79 (0.33)	0.32 (0.14–0.82)	0.29 (0.11–0.65)	358 (126)	360 (115)

CYP2C19*2 (681 G > A); rs4244285								
CYP2C19*1/*1 (*n* = 30)	0.58 (0.10)	0.60 (0.12)	0.74 (0.28)	0.76 (0.33)	0.32 (0.16–0.65)	0.31 (0.17–0.57)	382 (153)	382 (133)
CYP2C19*1/*2 (*n* = 19)	0.62 (0.13)	0.59 (0.19)	0.85 (0.31)	0.78 (0.33)	0.31 (0.14–0.67)	0.28 (0.14–0.57)	323 (66)	331 (78)

***P2Y12 H2***								
*P2Y12 H1/H1* (*n* = 33)	0.61 (0.12)	0.62 (0.15)	0.78 (0.31)	0.79 (0.35)	0.30 (0.16–0.57)	0.32 (0.16–0.65)	376 (150)	378 (131)
*P2Y12H1/H2* & *H2/H2* (*n* = 16)	0.58 (0.08)	0.54 (0.12)	0.80 (0.26)	0.70 (0.26)	0.34 (0.14–0.82)	0.24 (0.15–0.40) 2	323 (49)	327 (68)

1 Arithmetic mean (SD) or Geometric mean (range of 1 SD);

2 Significant interaction between 7-day changes in CRP concentration and P2Y12 (*p* = 0.038).

**Table 4:** Variation of inflammatory marker levels 1 in response to clopidogrel in function of platelet activity.

Platelet aggregation	Orosomucoid (g/L)		Haptoglobin (g/L)		CRP (mg/L)		CD40 (ng/L)	
			
	Day 1	Day 7	Day 1	Day 7	Day 1	Day 7	Day 1	Day 7
Responders (*n* = 32)	0.58 (0.10)	0.60 (0.12)	0.74 (0.28)	0.76 (0.33)	0.32 (0.16–0.65)	0.31 (0.17–0.57)	360 (135)	379 (132)
Hypo-responders (*n* = 17)	0.65 (0.12)	0.64 (0.19)	0.85 (0.31)	0.78 (0.33)	0.31 (0.14–0.67)	0.28 (0.14–0.57)	366 (132)	341 (78)

1 Arithmetic mean (SD) or Geometric mean (range of 1 SD).

**Table 5:** Genotyping data.

SNP (allele); db SNPAccession no.	Primers used for sequencing
CYP2C8	
*3 (1196 A > G); rs10509681	F: B-5′-GTGTTCTCCCAGTTTCTGCCC-3′ R: 5′-GACGCAGAGTAGAGTCACCCAC-3′
CYP2C9	
*2 (430 C > T); rs1799853	F: 5′-GTATTTTGGCCTGAAACCCATA-3′ R: B-5′-CACCCTTGGTTTTTCTCAACTC-3′
*3 (1075 A > C); rs1057910	F: B-5′-TGCACGAGGTCCAGAGAT-3′ R: 5′-GATACTATGAATTTGGGACTTC-3′
CYP2C19	
*2 (681 G > A); rs4244285	F: 5′-CAGAGCTTGGCATATTGTATC-3′ R: 5′-GTAAACACACAAAACTAGTCAATG-3′
CYP2J2	
*7(−50 G > A); rs890293	F: 5′-CATAGGAGAGACGGTGATTGAACC-3′ R: 5′-GGCGTCTTCTCGTCCTCCTGCA-3′
P2Y12	
*H2* Haplotype	As described in reference [33]
